# Goodbye work-related musculoskeletal disorders, welcome musculoskeletal health! A call for action

**DOI:** 10.5271/sjweh.4304

**Published:** 2026-05-01

**Authors:** P Paul FM Kuijer, Pieter Coenen

**Affiliations:** 1 Amsterdam UMC, Department of Public and Occupational Health, Amsterdam, The Netherlands.; 2 Amsterdam Public Health, Societal Participation & Health, Quality of Care, Amsterdam, The Netherlands.; 3 Amsterdam Movement Sciences, Musculoskeletal Health, Sports, Amsterdam, The Netherlands.

Despite decades of global attention, work-related musculoskeletal disorders (MSD) remain a significant challenge, with substantial personal and societal costs (1). The lack of progress in addressing this prevalent problem is not due to a lack of trying. Legislation and campaigns aimed at primary prevention of work-related MSD have been available in numerous countries (1). In an editorial written already about two decades ago, Wells (2) addressed the question "Why have we not solved the (work-related) MSD problem?". Formulated in six questions, Wells established the key factors to assess our ability to prevent work-related MSD: (i) How well do we understand MSD and their burdens?; (ii) How good are our MSD risk factors?; (iii) How effective and informative are current workplace MSD assessment approaches?; (iv) How effective are the recommended interventions in actually reducing MSD in the workplace?; (v) How intensely and widely implemented are workplace interventions to prevent MSD?; and (vi) How well are we improving disability outcomes for MSD?

In our paper on 50 years of research published in the *Scandinavian Journal of Work, Environment & Health* on MSD, it became clear that the first three questions have been sufficiently addressed to move forward (1). We have strong evidence on mechanisms as to how work-related MSD can develop and how they impact the lives of workers and society. In addition, there is an array of biopsychosocial risk factors for which we have evidence regarding the strength of their association with MSD. And, despite their limitations, we have various interview, questionnaire, observational and device-based approaches to determine exposures when assessing the work-relatedness of MSD.

Yet, there is a scarcity of good and well-implemented interventions to prevent work-related MSD. The CoWork (Copenhagen Work-related) musculoskeletal health model, as presented in this issue of the Journal (3), comes at an opportune time as it addresses the final three questions in Wells’ editorial (2). The authors of the CoWork musculoskeletal health model propose a paradigm shift (3). In short, the CoWork musculoskeletal health model suggests going from work-related biomechanical risk reduction only to adopting a biopsychosocial model for musculoskeletal health promotion at work. Musculoskeletal health is promoted using five integrated elements: (i) a health-oriented approach, (ii) a just-right work-related factor conceptualization, (iii) the Organizational, Management, Group, !ndividual (OMG!) workplace framework, (iv) an intervention guidance, and (v) a health economics perspective. The model provides guidance for all relevant actors – researchers, policymakers, practitioners, employers, and workers – to promote musculoskeletal health at the workplace. We commend the authors for coming up with the model!

What we like most about the CoWork musculoskeletal health model is that it explicitly addresses the positive health benefits of physical activity at work while not neglecting the negative effects by using ‘the just right principle’ (not too much and not too little). Practical examples of this positive benefit and the 'just right' principle are based on the authors’ studies on the benefits of micro-exercises (or exercise snacks) (4) and on interventions from the Goldilocks principle (5). In addition, we believe that the focus on supporting the good (musculoskeletal health) instead of preventing the bad (MSD) is probably more motivating for decision- and policy-makers to implement health-enhancing measures. To bring the CoWork musculoskeletal health model forward in science and practice, we make a call for action based on the following three considerations.

## A musculoskeletal health definition and measurement tool for better tailoring and evaluation

First, although musculoskeletal health is not a completely new concept (1), as far as we are aware, no agreed-upon definition and measurement tool exists. Such a definition and measurement tool are essential to assess the personal features of a worker to tailor a CoWork musculoskeletal health intervention. A good measurement tool is also needed to evaluate the effectiveness of CoWork musculoskeletal health interventions. The CoWork musculoskeletal health authors propose the following definition: "Work-related musculoskeletal health is a state of physical, mental, and social well-being of the locomotor (musculoskeletal) system in relation to work." This definition not just captures the musculoskeletal system but also incorporates important factors like fatigue, fitness, and functioning that play an important role in musculoskeletal health. Yet, reaching an international consensus on the definition of musculoskeletal health is essential – ideally involving all stakeholders – for example through a Delphi study. Next, a measurement tool that captures work-related musculoskeletal health can be developed, preferably based on current instruments to make comparisons with existing findings possible.

## A community of professionals for better intervention implementation

Second, the CoWork musculoskeletal health model is specifically designed for both research and practice. This brings up the question: ‘What specific competencies are needed for a professional to deliver a CoWork musculoskeletal health intervention?’ As the CoWork musculoskeletal health model is comprehensive and complex, incorporating elements of the full biopsychosocial perspective, effective implementation requires a truly multidisciplinary professional team. Therefore, a training program seems essential to build a community of CoWork musculoskeletal health professionals. A successful blueprint for such a program might be the internationally renowned Work Disability Prevention Canadian Institutes of Health Research Strategic Training Program (6). Key features of this program include participation from around the world, community building through yearly consecutive two-week training sessions, multidisciplinary developers of the training program acting as mentors, recognized guest speakers from around the world, e-courses, and, of course, the active involvement of stakeholders like employers, unions, and workers’ compensation boards. In Europe, perhaps the Marie-Sklodowska Curie actions might provide financial support for such a program, given the proposed excellence in research and innovation for doctoral and postdoctoral training. This would also contribute to community building and establishing a strong research field.

## Making it work: combining health promotion and risk factor prevention for blue-collar workers

Finally, promoting work-related musculoskeletal health by stimulating health-enhancing factors such as physical activity can be effective, but this is easier said than done (7, 8). We know from the literature that the effectiveness of workplace health promotion is moderate at best (9). The ‘just-right’ work, as advocated in the CoWork paper, is mostly supported by the implementation of exercise snacks (4), while there is limited evidence on the effectiveness of the Goldilocks principle yet (5). Also, interventions on reducing physical workload have shown limited effectiveness (10). Although these interventions show slender effectiveness as standalone measures to either promote health or reduce workload, combining effective elements from these two groups of interventions could be the way forward. This is also theoretically plausible as work, health, and lifestyle are strongly intertwined. As an example, we may not be able to improve health-enhancing physical activity after work without actually doing something about work, since it is known that physical work demands prevent workers from being active during their leisure time (11). The latter is particularly important for workers who should theoretically have the most potential to benefit from the CoWork musculoskeletal health model, namely blue-collar workers in physically demanding jobs. Yet, worksite health promotion and improvement programs among blue-collar workers do not often live up to their potential (9, 12). This is despite the fact that work itself accounts for a large part of the musculoskeletal health of blue-collar workers in physically demanding jobs. For example, the population attributable fraction (PAF) of physical work demands show that they account for 7–10% of low-back pain and 15–25% of lateral epicondylitis among the Dutch workforce (13). Among Dutch blue-collar floor layers, the PAF ranges from 35% for knee osteoarthritis to 55% for lumbosacral radicular syndrome (14). Therefore, we ask CoWorkers to ensure that blue-collar workers are well served in CoWork musculoskeletal health interventions, since there is a large potential for such interventions to reduce health inequalities.

In addition, research on health promotion – though not always focused on the workplace – shows that interventions are most effective when they move beyond individual-level programs to address the system drivers of unhealthy behaviors (15–17). This literature demonstrates that a systems approach helps explain why unhealthy patterns persist by highlighting issues such as coordination, learning processes, and structural barriers that interventions must address to achieve meaningful impact. Evidence shows that multilevel strategies that reshape organizational culture and job design – and do so with environmental support – could produce more durable lifestyle improvements (15–17). As such, a multifaceted approach that simultaneously stimulates the good (health enhancing work factors) and prevents the bad (strenuous physical work demands) is likely to be most effective. Sundstrup and colleagues (18) presented inspiring examples regarding combining ergonomics, physical exercise programs, and multifaceted strategies at the workplace.

In summary, the CoWork musculoskeletal health model has the potential to facilitate research and practice across disciplines and make a difference for workers’ musculoskeletal health. To do so, a clear definition of musculoskeletal health – along with reliable and validated measurement instruments and an engaged community of professionals – is essential for the successful implementation, testing, and advancement of the model. Thereby, the model might contribute to a better understanding of how to effectively promote, initiate, develop, implement, and evaluate interventions to enhance musculoskeletal health, including, of course, prevention of MSD. By doing so, we are confident that the final three questions of Wells’s editorial will be sufficiently addressed in forthcoming decades.
